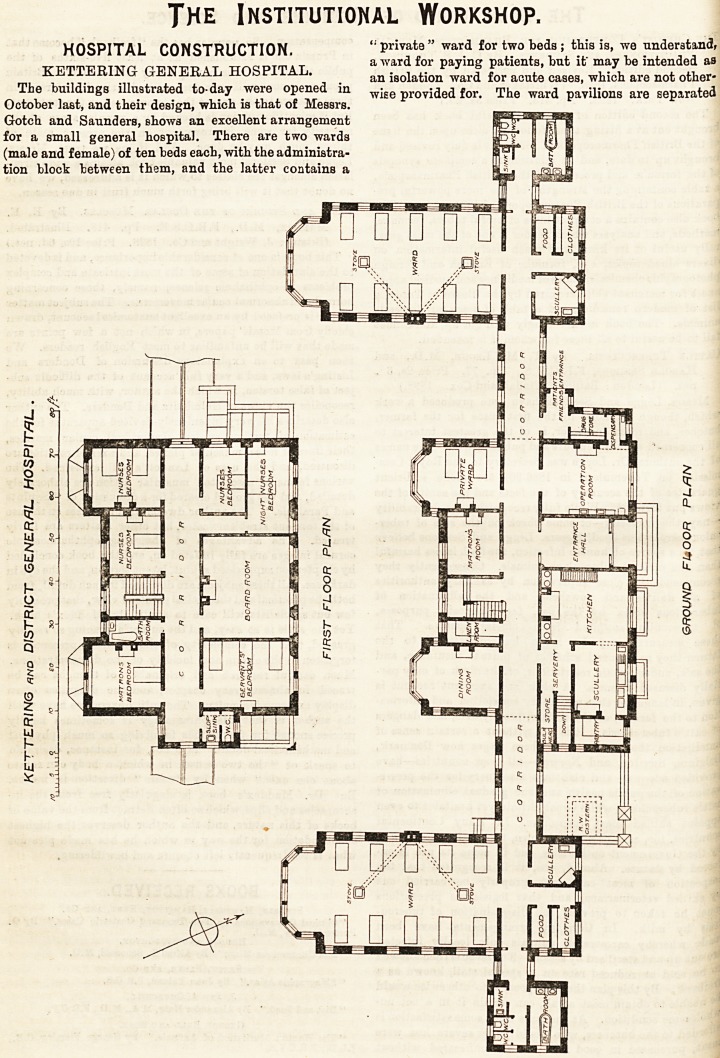# Hospital Construction

**Published:** 1898-10-08

**Authors:** 


					36 THE HOSPITAL. Oct. 8, 1898.
HOSPITAL CONSTRUCTION.
KETTERING GENERAL HOSPITAL.
The buildings illustrated to-day were opened in
October last, and their design, which is that of Messrs.
Gotch and Saunders, shows an excellent arrangement
for a small general hospital. There are two wards
(male and female) of ten beds each, with the administra-
tion block between them, and the latter contains a
" private " ward for two beds ; this is, we understand
a ward for paying patients, but it' may be intended as
an isolation ward for acute cases, which are not other-
wise provided for. The ward pavilions are separated
The Institutional Workshop.
HOSPITAL CONSTRUCTION. "private" ward for two beds ; this is, we understand*
T award for paving patients, but it' may be intended as
KETTERING GENERAL HOSP . an j80iati0n ward for acute cases, which are not other-
The buildings illustrated to-day were opened in wjge provided for. The ward pavilions are separated
October last, and their design, which is that of Messrs.
Gotch and Saunders, shows an excellent arrangement
for a small general hospital. There are two wards
(male and female) of ten beds each, with the administra-
tion block between them, and the latter contains a
O
to
Q
Q
?
tc
I
?
Ul
f-
h-
Ui
sc
?
5 ? HI-^i ff Is B 15 *
. ?? o A . i L jt4 i ^ IL s1
J \ 1 ft ib_ w *?> ta 1 lA 1 o E3?n ^
XJBMW I'll 1 Tjmrm M ? < HJ W FH ?T^jnii ji ?? U i r=n   . |?I PSf
j j i ?;-_i .' ii s a -r ' f . <"
s' ! IM: f 1 si | p g< ] ?
t8 Hf - h ! I ? rr" q
C IW li ill1. q ;1 ? ?.MM] 4a J <1 "I o
h1 8" fflol SI ^ * (II b R t yj
&
0cT- 8> 1S98- THE HOSPITAL. 37
from the central block by well-appointed corridors, and
are each furnished with, a scullery, larder, and room
for patients' clotbes ; while the sinks, w.c.'s, baths, and
lavatories are accommodated in small blocks on the
further side, separated by cross-ventilated passages.
The wards run nearly north and south, have ample light
and ventilation at each side, and at the south end have a
large bay, wbich must add greatly to the cheerfulness
of the interior, besides being useful for patients wbo
can leave their beds. The arrangements of the adminis-
trative block (which is two-storied) are sufficiently
shown by the plans, the only points requiring comment
being that the dispensary, which appears to be entered
through the operating-room, is extremely small, and is
connected with a drug 6tore, which is also unusually
restricted in size. The upper floor contains tbe bed-
rooms for the staff, but it does not appear that there is
a BittiDg-room for the nurses. The ward floors are of
pitch pine, wax-polished, and the corridor floors of
terrazzo. The walls internally are finished with cement,
painted ; and externally, are faced with a brown local
stone, wbile the roof is of red tiles. The internal fit-
tings appear to be suitable and complete, and the
hospital is one which should prove of great service to
the district in which it stands. The cost is stated
(without furnishing or fittings) at about ?6,000, or ?500
per bed.

				

## Figures and Tables

**Figure f1:**